# Chlorido{*N*-[2-(diphenyl­phosphan­yl)benz­yl]-1-(pyridin-2-yl)methanamine-κ*P*}gold(I)

**DOI:** 10.1107/S1600536811044205

**Published:** 2011-10-29

**Authors:** Telisha Traut, Frederik H. Kriel, Werner E. van Zyl, D. Bradley G. Williams

**Affiliations:** a Department of Chemistry, University of Johannesburg, PO Box 524, Auckland Park 2006, South Africa; b Biomed, Mintek, Private Bag X3015, Randburg 2125, South Africa

## Abstract

In the title compound, [AuCl(C_25_H_23_N_2_P)], the Au^I^ atom is in a typical almost linear coordination environment defined by phosphane P and Cl atoms [bond angle = 175.48 (4)°]. Helical supra­molecular chains along the *b* axis and mediated by N—H⋯Cl hydrogen bonds feature in the crystal packing.

## Related literature

For previously published crystal structures of related *P*,*N-*type Au(I) complexes, see: Williams *et al.* (2007 ▶). For catalytic reactions of these types of complexes, see: Williams & Pretorius (2008 ▶). For related structures, see: Baenziger *et al.* (1976 ▶); Bellon *et al.* (1969 ▶). For the synthesis of the ligand, see: Shirakawa *et al.* (1997 ▶).
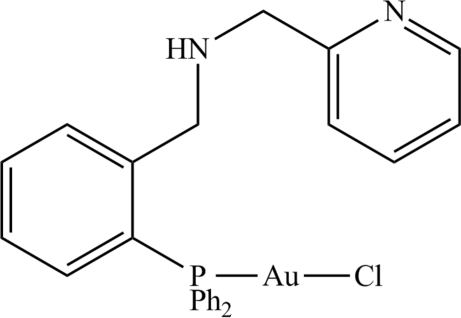


## Experimental

### 

#### Crystal data

[AuCl(C_25_H_23_N_2_P)]
*M* *_r_* = 614.84
Monoclinic, 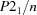

*a* = 12.5888 (9) Å
*b* = 14.1443 (10) Å
*c* = 13.2354 (11) Å
β = 107.128 (3)°
*V* = 2252.2 (3) Å^3^
*Z* = 4
Mo *K*α radiation
μ = 6.74 mm^−1^
*T* = 173 K
0.40 × 0.18 × 0.16 mm

#### Data collection

Bruker SMART CCD area-detector diffractometer
Absorption correction: integration (*SADABS*; Bruker, 1999 ▶) *T*_min_ = 0.303, *T*_max_ = 0.559
13864 measured reflections
5572 independent reflections
4399 reflections with *I* > 2σ(*I*)
*R*_int_ = 0.056

#### Refinement

*R*[*F* ^2^ > 2σ(*F* ^2^)] = 0.029
*wR*(*F* ^2^) = 0.067
*S* = 0.97
5572 reflections
275 parameters
H atoms treated by a mixture of independent and constrained refinement
Δρ_max_ = 1.96 e Å^−3^
Δρ_min_ = −1.30 e Å^−3^

### 

Data collection: *SMART* (Bruker, 1999 ▶); cell refinement: *SAINT* (Bruker, 1999 ▶); data reduction: *SAINT*; program(s) used to solve structure: *SHELXS97* (Sheldrick, 2008 ▶); program(s) used to refine structure: *SHELXL97* (Sheldrick, 2008 ▶); molecular graphics: *Mercury* (Macrae *et al.*, 2006 ▶); software used to prepare material for publication: *WinGX* (Farrugia, 1999 ▶).

## Supplementary Material

Crystal structure: contains datablock(s) I, global. DOI: 10.1107/S1600536811044205/tk5001sup1.cif

Structure factors: contains datablock(s) I. DOI: 10.1107/S1600536811044205/tk5001Isup2.hkl

Additional supplementary materials:  crystallographic information; 3D view; checkCIF report

## Figures and Tables

**Table 1:** Selected bond lengths (Å)

Au1—P1	2.2410 (10)
Au1—Cl1	2.2921 (10)

**Table 2:** Hydrogen-bond geometry (Å, °)

*D*—H⋯*A*	*D*—H	H⋯*A*	*D*⋯*A*	*D*—H⋯*A*
N1—H1⋯Cl1^i^	0.87 (6)	2.68 (5)	3.536 (4)	169 (5)

Symmetry code: (i) 
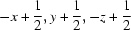
.

**Fig. 1.**
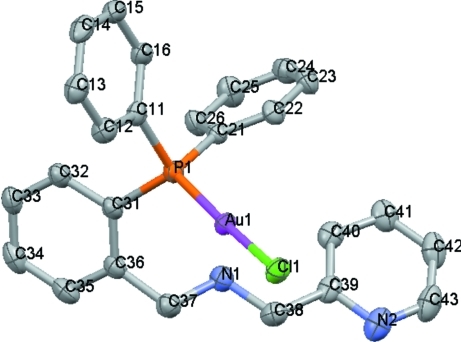
Molecular structure of the title compound drawn with dispacement ellipsoids at the 50% probability level. Hydrogen atoms have been omitted for clarity.

## supplementary crystallographic information

### Comment

We have previously published crystal structure data regarding three related *P*,*N* type Au(I) complexes, two of which exhibited inter- and intra-molecular gold—gold interactions, as well as displaying differential gold binding affinity for the phosphorus and nitrogen atoms, respectively (Williams *et al.*, 2007). These complexes were obtained as a part of our continued interest in the versatility and transition metal complexation of heteroditopic, multifunctional *P*,*N*-based ligands. We are especially interested in this class of compounds for their proven efficiency as catalysts in certain chemical reactions (Williams *et al.*, 2008), as well as for their potential in medicinal applications, an aspect which has not received much attention in literature..

As a part of this on-going study, we have prepared an amino-phosphine ligand (II) from 2-(diphenylphosphanyl)benzaldehyde as starting material (I), proceeding *via* the Schiff base which is reduced to amine (II). This *P*,*N* product formed the crystalline title Au(I) complex (III) from a saturated chloroform solution. This complex is of particular interest as the two N atoms should in theory have different gold binding affinities due to the difference in the state of their hybridization (*sp*^2^*vs sp*^3^), in keeping with previously published results (Williams, *et al.*, 2007).

The title compound (III), Fig. 1, crystallizes in the monoclinic space group *P*2_1_*/c.* The crystal packing is stabilized by weak intra-molecular N—H···Cl interactions (Fig. 2). The coordination at the gold metal centre showed a virtually linear P—Au—Cl system (bond angle of 175.48 (4)°) as is common for two-coordinate Au(I) compounds. The Au—P distance of 2.241 (1) Å compares favourably to the Au—P distance in the similar (triphenylphosphine)gold(I) chloride structure of 2.235 (3) Å (Baenziger *et al.*, 1976), but is shorter than the corresponding bond distance in the related (triphenylphosphine)gold(I) cyanide of 2.27 (1) Å (Bellon *et al.*, 1969). As expected, the Au—Cl distance of 2.292 (1) Å compares well with that observed for (triphenylphosphine)gold(I) chloride at 2.279 (3) Å.

### Experimental

The ligand (II) was synthesized in a similar manner to a literature procedure (Shirakawa *et al.*, 1997) and the title compound was prepared as per previously described methods (Williams *et al.*, 2007).

The amino-phosphine ligand (II) employed in this study was prepared from the 2-(diphenylphosphanyl)benzaldehyde starting material (I). The synthesis involved reacting (I) (300 mg, 0.689 mmol) with 1.5 equivalents of 2-(aminomethyl)pyridine (0.106 ml, 1.033 mmol) in toluene (15 ml) as solvent. The reaction mixture was stirred under reflux (oil bath temperature 140–150 °C) for 5 h, after which the solvent was removed *in vacuo*. The intermediate imino-phosphine product was dissolved in dried MeOH (10 ml). NaBH_4_ (3 equivalents) was added and the reaction mixture stirred at room temperature for 15 h. The reduction reaction was quenched by the addition of deionized H_2_O, and the mixture was extracted with H_2_O and DCM and the resultant organic phase dried over Na_2_SO_4_. Solids were removed *via* filtration and the solvent removed *in vacuo*. The pure amino-phosphine ligand (II) was recovered in high yield (85%) after bulb-to-bulb vacuum distillation.

Amino-phosphine ligand (II) (120 mg, 0.314 mmol) was dissolved in 20 mL of diethyl ether. To this solution were added 0.95 equivalents of (THT)AuCl (96 mg, 0.298 mmol) dissolved in 2 mL of chloroform. The (THT)AuCl solution was slowly added to the ligand solution and the mixture stirred at room temperature for 5 minutes. The solvent was evaporated *in vacuo* to *ca* 5 ml and the white, powdered product (III) was precipitated from the solution by the addition of 10 ml cold hexane (x3). Colourless monoclinic crystals were grown from a chloroform solution of the product. Yield 108 mg, 56%. ^1^H NMR (CDCl_3_, 300 MHz, p.p.m.) 8.41 [d, *J* = 4.5 Hz, 1H, aromatic], 7.64 [dd, *J* = 6.6 and 5.4 Hz, 1H, aromatic], 7.57–7.36 [m, 11H, aromatic], 7.20 [d, *J* = 7.2 Hz, 2H, aromatic], 7.13–7.06 [m, 2H, aromatic], 6.81 [dd, *J* = 12.8 and 4.1 Hz, 1H, aromatic], 4.20 [s, 2H, Ar—CH_2_N], 3.68 [s, 2H, NCH_2_], 1.81 [s, NH]. ^13^C{^1^H} NMR: (CDCl_3_, 75 MHz, p.p.m.) 158.8 [s, 1 C], 149.1 [s, 1 C], 144.1 [d, *J_C,P_* = 10.9 Hz, 1 C], 136.4 [s, 1 C], 134.1 [d, *J_C,P_* = 13.8 Hz, 4 C], 133.9 [d, *J_C,P_* = 7.1 Hz, 1 C], 131.8 [d, *J_C,P_* = 2.3 Hz, 1 C], 131.6 [d, *J_C,P_* = 2.3 Hz, 2 C], 130.3 [d, *J_C,P_* = 8.9 Hz, 1 C], 129.8 [s, 1 C], 129.1 [d, *J_C,P_* = 11.8 Hz, 4 C], 128.1 [d, *J_C,P_* = 16.3 Hz, 1 C], 127.3 [d, *J_C,P_* = 10.0 Hz, 1 C], 126.7 [d, *J_C,P_* = 59.0 Hz, 1 C], 122.4 [s, 1 C], 121.8 [s, 1 C], 54.1 [s, 1 C], 52.5 [d, *J_C,P_* = 16.3 Hz, 1 C]. ^31^P{^1^H} NMR (CDCl_3_, 121.42 MHz, p.p.m.): 26.7 [*s*].

### Refinement

The H atoms were positioned geometrically and allowed to ride on their respective parent atoms, with C—H = 0.93 (Ar—H) or 0.97 (CH_2_) Å, and with *U*_eq_ = 1.2*U*_eq_(C). The amine-H atom was refined. The maximum and minimum residual electron density peaks of 1.96 and 1.30 eÅ^-3^, respectively, were located 0.88 Å and 0.76 Å from the Au1 atom.

### Crystal data

**Table:**

[AuCl(C_25_H_23_N_2_P)]	*Z* = 4
*M**_r_* = 614.84	*F*(000) = 1192
Monoclinic, *P*2_1_/*n*	*D*_x_ = 1.813 Mg m^−^^3^
Hall symbol: -P 2yn	Mo *K*α radiation, λ = 0.71073 Å
*a* = 12.5888 (9) Å	µ = 6.74 mm^−^^1^
*b* = 14.1443 (10) Å	*T* = 173 K
*c* = 13.2354 (11) Å	Needle, colourless
β = 107.128 (3)°	0.40 × 0.18 × 0.16 mm
*V* = 2252.2 (3) Å^3^	

### Data collection

**Table:**

Bruker SMART CCD area-detector diffractometer	5572 independent reflections
Radiation source: sealed tube	4399 reflections with *I* > 2σ(*I*)
graphite	*R*_int_ = 0.056
phi and ω scans	θ_max_ = 28.3°, θ_min_ = 2.0°
Absorption correction: integration (*SADABS*; Bruker, 1999)	*h* = −15→16
*T*_min_ = 0.303, *T*_max_ = 0.559	*k* = −14→18
13864 measured reflections	*l* = −17→13

### Refinement

**Table:**

Refinement on *F*^2^	Primary atom site location: structure-invariant direct methods
Least-squares matrix: full	Secondary atom site location: difference Fourier map
*R*[*F*^2^ > 2σ(*F*^2^)] = 0.029	Hydrogen site location: inferred from neighbouring sites
*wR*(*F*^2^) = 0.067	H atoms treated by a mixture of independent and constrained refinement
*S* = 0.97	*w* = 1/[σ^2^(*F*_o_^2^) + (0.0307*P*)^2^] where *P* = (*F*_o_^2^ + 2*F*_c_^2^)/3
5572 reflections	(Δ/σ)_max_ = 0.001
275 parameters	Δρ_max_ = 1.96 e Å^−^^3^
0 restraints	Δρ_min_ = −1.30 e Å^−^^3^

### Special details

**Table:**

Geometry. All e.s.d.'s (except the e.s.d. in the dihedral angle between two l.s. planes) are estimated using the full covariance matrix. The cell e.s.d.'s are taken into account individually in the estimation of e.s.d.'s in distances, angles and torsion angles; correlations between e.s.d.'s in cell parameters are only used when they are defined by crystal symmetry. An approximate (isotropic) treatment of cell e.s.d.'s is used for estimating e.s.d.'s involving l.s. planes.
Refinement. Refinement of *F*^2^ against ALL reflections. The weighted *R*-factor *wR* and goodness of fit *S* are based on *F*^2^, conventional *R*-factors *R* are based on *F*, with *F* set to zero for negative *F*^2^. The threshold expression of *F*^2^ > σ(*F*^2^) is used only for calculating *R*-factors(gt) *etc*. and is not relevant to the choice of reflections for refinement. *R*-factors based on *F*^2^ are statistically about twice as large as those based on *F*, and *R*- factors based on ALL data will be even larger.

### Fractional atomic coordinates and isotropic or equivalent isotropic displacement parameters (Å^2^)

**Table:**

	*x*	*y*	*z*	*U*_iso_*/*U*_eq_	
C11	0.3621 (3)	0.3261 (3)	−0.0026 (3)	0.0274 (8)	
C12	0.3958 (3)	0.2313 (3)	0.0098 (4)	0.0334 (9)	
H12	0.3973	0.1994	0.0716	0.040*	
C13	0.4266 (3)	0.1849 (3)	−0.0686 (4)	0.0394 (10)	
H13	0.4504	0.1224	−0.0591	0.047*	
C14	0.4222 (3)	0.2317 (3)	−0.1619 (4)	0.0395 (11)	
H14	0.4421	0.2001	−0.2152	0.047*	
C15	0.3883 (3)	0.3247 (4)	−0.1758 (3)	0.0374 (10)	
H15	0.3854	0.3558	−0.2384	0.045*	
C16	0.3585 (3)	0.3719 (3)	−0.0958 (3)	0.0323 (9)	
H16	0.3360	0.4347	−0.1052	0.039*	
C21	0.2684 (3)	0.4968 (3)	0.0550 (3)	0.0247 (8)	
C22	0.1574 (3)	0.5053 (3)	−0.0094 (3)	0.0304 (9)	
H22	0.1113	0.4525	−0.0237	0.036*	
C23	0.1170 (3)	0.5929 (3)	−0.0515 (3)	0.0373 (10)	
H23	0.0441	0.5982	−0.0945	0.045*	
C24	0.1844 (4)	0.6721 (3)	−0.0297 (3)	0.0376 (10)	
H24	0.1567	0.7305	−0.0578	0.045*	
C25	0.2925 (3)	0.6644 (3)	0.0336 (4)	0.0396 (11)	
H25	0.3376	0.7178	0.0482	0.048*	
C26	0.3350 (3)	0.5775 (3)	0.0758 (3)	0.0330 (9)	
H26	0.4083	0.5731	0.1183	0.040*	
C31	0.4514 (3)	0.3993 (3)	0.2079 (3)	0.0257 (8)	
C32	0.5495 (3)	0.4040 (3)	0.1776 (3)	0.0313 (9)	
H32	0.5454	0.3985	0.1065	0.038*	
C33	0.6517 (3)	0.4168 (3)	0.2519 (4)	0.0348 (10)	
H33	0.7159	0.4200	0.2308	0.042*	
C34	0.6584 (3)	0.4249 (3)	0.3571 (4)	0.0414 (11)	
H34	0.7273	0.4333	0.4070	0.050*	
C35	0.5637 (3)	0.4205 (3)	0.3893 (4)	0.0380 (10)	
H35	0.5694	0.4270	0.4606	0.046*	
C36	0.4582 (3)	0.4061 (3)	0.3152 (3)	0.0289 (9)	
C37	0.3585 (3)	0.3989 (3)	0.3553 (3)	0.0337 (9)	
H37A	0.3271	0.3359	0.3413	0.040*	
H37B	0.3814	0.4087	0.4312	0.040*	
C38	0.1744 (3)	0.4593 (3)	0.3383 (3)	0.0351 (9)	
H38A	0.1951	0.4629	0.4148	0.042*	
H38B	0.1420	0.3974	0.3175	0.042*	
C39	0.0880 (3)	0.5339 (3)	0.2923 (3)	0.0322 (9)	
C40	0.0988 (3)	0.5991 (3)	0.2179 (3)	0.0332 (9)	
H40	0.1613	0.5989	0.1940	0.040*	
C41	0.0137 (3)	0.6655 (3)	0.1792 (4)	0.0402 (11)	
H41	0.0190	0.7098	0.1290	0.048*	
C42	−0.0769 (3)	0.6645 (3)	0.2160 (4)	0.0444 (12)	
H42	−0.1338	0.7084	0.1920	0.053*	
C43	−0.0821 (4)	0.5968 (4)	0.2897 (4)	0.0489 (13)	
H43	−0.1446	0.5961	0.3137	0.059*	
P1	0.31973 (7)	0.38097 (7)	0.10429 (8)	0.0245 (2)	
Cl1	0.06264 (8)	0.17656 (7)	0.15389 (8)	0.0337 (2)	
Au1	0.193361 (11)	0.282466 (10)	0.135160 (12)	0.02541 (5)	
N1	0.2738 (3)	0.4689 (3)	0.3046 (3)	0.0327 (8)	
N2	−0.0018 (3)	0.5310 (3)	0.3295 (3)	0.0449 (10)	
H1	0.307 (4)	0.523 (4)	0.319 (4)	0.045 (14)*	

### Atomic displacement parameters (Å^2^)

**Table:**

	*U*^11^	*U*^22^	*U*^33^	*U*^12^	*U*^13^	*U*^23^
C11	0.0214 (16)	0.028 (2)	0.032 (2)	−0.0037 (15)	0.0072 (15)	−0.0029 (18)
C12	0.039 (2)	0.028 (2)	0.036 (2)	0.0002 (16)	0.0145 (18)	−0.0009 (18)
C13	0.036 (2)	0.034 (2)	0.050 (3)	−0.0009 (18)	0.016 (2)	−0.007 (2)
C14	0.033 (2)	0.051 (3)	0.038 (3)	−0.0051 (19)	0.0151 (18)	−0.017 (2)
C15	0.031 (2)	0.053 (3)	0.029 (2)	0.0008 (19)	0.0112 (17)	0.002 (2)
C16	0.0264 (19)	0.037 (2)	0.034 (2)	0.0003 (16)	0.0101 (17)	0.0055 (19)
C21	0.0259 (17)	0.025 (2)	0.022 (2)	0.0030 (14)	0.0057 (15)	0.0003 (16)
C22	0.0282 (18)	0.031 (2)	0.029 (2)	−0.0028 (16)	0.0026 (16)	−0.0034 (18)
C23	0.030 (2)	0.041 (3)	0.035 (2)	0.0055 (18)	0.0004 (17)	0.002 (2)
C24	0.044 (2)	0.031 (2)	0.036 (3)	0.0088 (19)	0.0098 (19)	0.005 (2)
C25	0.038 (2)	0.025 (2)	0.053 (3)	−0.0047 (17)	0.008 (2)	0.002 (2)
C26	0.0251 (18)	0.033 (2)	0.035 (2)	−0.0007 (16)	0.0003 (16)	0.0041 (19)
C31	0.0264 (17)	0.022 (2)	0.027 (2)	0.0022 (14)	0.0049 (15)	0.0038 (16)
C32	0.0315 (19)	0.028 (2)	0.032 (2)	0.0012 (16)	0.0061 (17)	0.0045 (18)
C33	0.0224 (17)	0.035 (2)	0.044 (3)	0.0026 (16)	0.0042 (17)	0.006 (2)
C34	0.030 (2)	0.037 (3)	0.047 (3)	0.0009 (17)	−0.0056 (19)	0.002 (2)
C35	0.041 (2)	0.036 (3)	0.029 (2)	0.0055 (18)	−0.0014 (18)	0.003 (2)
C36	0.0312 (19)	0.025 (2)	0.029 (2)	0.0053 (15)	0.0056 (16)	0.0025 (17)
C37	0.037 (2)	0.031 (2)	0.033 (2)	0.0028 (17)	0.0109 (18)	0.0035 (19)
C38	0.040 (2)	0.034 (2)	0.033 (2)	−0.0014 (18)	0.0140 (18)	0.0005 (19)
C39	0.0318 (19)	0.028 (2)	0.038 (2)	−0.0055 (16)	0.0120 (18)	−0.0112 (19)
C40	0.034 (2)	0.028 (2)	0.038 (2)	−0.0069 (17)	0.0109 (18)	−0.0106 (19)
C41	0.037 (2)	0.032 (3)	0.047 (3)	−0.0057 (18)	0.004 (2)	−0.009 (2)
C42	0.031 (2)	0.035 (3)	0.061 (3)	−0.0015 (18)	0.004 (2)	−0.015 (2)
C43	0.033 (2)	0.047 (3)	0.070 (4)	−0.007 (2)	0.020 (2)	−0.017 (3)
P1	0.0234 (4)	0.0238 (5)	0.0260 (5)	0.0000 (4)	0.0068 (4)	0.0013 (4)
Cl1	0.0320 (5)	0.0304 (5)	0.0425 (6)	−0.0029 (4)	0.0168 (4)	0.0038 (5)
Au1	0.02563 (8)	0.02361 (8)	0.02855 (9)	−0.00060 (6)	0.01040 (6)	0.00008 (6)
N1	0.0344 (17)	0.027 (2)	0.036 (2)	0.0019 (15)	0.0107 (15)	0.0005 (16)
N2	0.042 (2)	0.035 (2)	0.062 (3)	−0.0060 (17)	0.0234 (19)	−0.011 (2)

### Geometric parameters (Å, °)

**Table:**

C11—C16	1.383 (6)	C32—H32	0.9300
C11—C12	1.401 (6)	C33—C34	1.375 (6)
C11—P1	1.825 (4)	C33—H33	0.9300
C12—C13	1.377 (6)	C34—C35	1.381 (6)
C12—H12	0.9300	C34—H34	0.9300
C13—C14	1.387 (7)	C35—C36	1.414 (5)
C13—H13	0.9300	C35—H35	0.9300
C14—C15	1.378 (7)	C36—C37	1.503 (6)
C14—H14	0.9300	C37—N1	1.465 (5)
C15—C16	1.394 (6)	C37—H37A	0.9700
C15—H15	0.9300	C37—H37B	0.9700
C16—H16	0.9300	C38—N1	1.454 (5)
C21—C26	1.394 (5)	C38—C39	1.508 (6)
C21—C22	1.412 (5)	C38—H38A	0.9700
C21—P1	1.811 (4)	C38—H38B	0.9700
C22—C23	1.391 (6)	C39—N2	1.360 (5)
C22—H22	0.9300	C39—C40	1.385 (6)
C23—C24	1.384 (6)	C40—C41	1.402 (6)
C23—H23	0.9300	C40—H40	0.9300
C24—C25	1.377 (6)	C41—C42	1.366 (6)
C24—H24	0.9300	C41—H41	0.9300
C25—C26	1.390 (6)	C42—C43	1.382 (7)
C25—H25	0.9300	C42—H42	0.9300
C26—H26	0.9300	C43—N2	1.361 (6)
C31—C36	1.400 (6)	C43—H43	0.9300
C31—C32	1.407 (5)	Au1—P1	2.2410 (10)
C31—P1	1.832 (4)	Au1—Cl1	2.2921 (10)
C32—C33	1.383 (5)	N1—H1	0.86 (5)
			
C16—C11—C12	118.8 (4)	C33—C34—H34	119.7
C16—C11—P1	123.5 (3)	C35—C34—H34	119.7
C12—C11—P1	117.7 (3)	C34—C35—C36	120.8 (4)
C13—C12—C11	120.7 (4)	C34—C35—H35	119.6
C13—C12—H12	119.6	C36—C35—H35	119.6
C11—C12—H12	119.6	C31—C36—C35	118.6 (4)
C12—C13—C14	119.8 (4)	C31—C36—C37	123.0 (3)
C12—C13—H13	120.1	C35—C36—C37	118.4 (4)
C14—C13—H13	120.1	N1—C37—C36	111.3 (3)
C15—C14—C13	120.3 (4)	N1—C37—H37A	109.4
C15—C14—H14	119.9	C36—C37—H37A	109.4
C13—C14—H14	119.9	N1—C37—H37B	109.4
C14—C15—C16	119.8 (4)	C36—C37—H37B	109.4
C14—C15—H15	120.1	H37A—C37—H37B	108.0
C16—C15—H15	120.1	N1—C38—C39	113.2 (4)
C11—C16—C15	120.5 (4)	N1—C38—H38A	108.9
C11—C16—H16	119.7	C39—C38—H38A	108.9
C15—C16—H16	119.7	N1—C38—H38B	108.9
C26—C21—C22	118.7 (3)	C39—C38—H38B	108.9
C26—C21—P1	122.6 (3)	H38A—C38—H38B	107.7
C22—C21—P1	118.6 (3)	N2—C39—C40	122.9 (4)
C23—C22—C21	119.8 (4)	N2—C39—C38	114.2 (4)
C23—C22—H22	120.1	C40—C39—C38	122.9 (4)
C21—C22—H22	120.1	C39—C40—C41	118.8 (4)
C24—C23—C22	120.6 (4)	C39—C40—H40	120.6
C24—C23—H23	119.7	C41—C40—H40	120.6
C22—C23—H23	119.7	C42—C41—C40	119.4 (5)
C25—C24—C23	119.8 (4)	C42—C41—H41	120.3
C25—C24—H24	120.1	C40—C41—H41	120.3
C23—C24—H24	120.1	C41—C42—C43	118.5 (4)
C24—C25—C26	120.6 (4)	C41—C42—H42	120.8
C24—C25—H25	119.7	C43—C42—H42	120.8
C26—C25—H25	119.7	N2—C43—C42	124.2 (4)
C25—C26—C21	120.4 (3)	N2—C43—H43	117.9
C25—C26—H26	119.8	C42—C43—H43	117.9
C21—C26—H26	119.8	C21—P1—C11	105.08 (18)
C36—C31—C32	119.3 (3)	C21—P1—C31	106.85 (17)
C36—C31—P1	122.7 (3)	C11—P1—C31	103.51 (17)
C32—C31—P1	118.0 (3)	C21—P1—Au1	115.56 (12)
C33—C32—C31	121.0 (4)	C11—P1—Au1	105.18 (13)
C33—C32—H32	119.5	C31—P1—Au1	119.07 (13)
C31—C32—H32	119.5	P1—Au1—Cl1	175.48 (4)
C34—C33—C32	119.9 (4)	C38—N1—C37	111.9 (3)
C34—C33—H33	120.1	C38—N1—H1	115 (3)
C32—C33—H33	120.1	C37—N1—H1	105 (3)
C33—C34—C35	120.5 (4)	C39—N2—C43	116.2 (4)

### Hydrogen-bond geometry (Å, °)

**Table:**

*D*—H···*A*	*D*—H	H···*A*	*D*···*A*	*D*—H···*A*
N1—H1···Cl1^i^	0.87 (6)	2.68 (5)	3.536 (4)	169 (5)

Symmetry codes: (i) −*x*+1/2, *y*+1/2, −*z*+1/2.
